# Interest of Absolute Eosinopenia as a Marker of Influenza in Outpatients during the Fall-Winter Seasons 2016–2018 in the Greater Paris Area: The SUPERFLUOUS Study

**DOI:** 10.3390/diagnostics13122115

**Published:** 2023-06-19

**Authors:** Benjamin Davido, Benoit Lemarie, Elyanne Gault, Jennifer Dumoulin, Emma D’anglejan, Sebastien Beaune, Pierre De Truchis

**Affiliations:** 1 Maladies Infectieuses, Hôpital Raymond Poincaré, Assistance Publique des Hôpitaux de Paris (AP-HP), 92380 Garches, France; benoitlemarie@orange.fr (B.L.); emma.danglejan@gmail.com (E.D.); p.de-truchis@aphp.fr (P.D.T.); 2 UMR1173, Université Versailles St-Quentin, Université Paris-Saclay, 78180 Montigny-Le-Bretonneux, France; elyanne.gault@aphp.fr; 3 Virologie, Hôpital Ambroise-Paré, Assistance Publique des Hôpitaux de Paris (AP-HP), 92100 Boulogne-Billancourt, France; 4 Pneumologie, Hôpital Ambroise Paré, Assistance Publique des Hôpitaux de Paris (AP-HP), 92100 Boulogne-Billancourt, France; jennifer.dumoulin@aphp.fr; 5 Service d’Accueil des Urgences, Hôpital Ambroise Paré, Assistance Publique des Hôpitaux de Paris (AP-HP), 92100 Boulogne-Billancourt, France; sebastien.beaune@aphp.fr

**Keywords:** eosinophil, respiratory tract infections, influenza virus, paramyxovirus

## Abstract

Introduction: Prior to the emergence of COVID-19, when influenza was the predominant cause of viral respiratory tract infections (VRTIs), this study aimed to analyze the distinct biological abnormalities associated with influenza in outpatient settings. Methods: A multicenter retrospective study was conducted among outpatients, with the majority seeking consultation at the emergency department, who tested positive for VRTIs using RT-PCR between 2016 and 2018. Patient characteristics were compared between influenza (A and B types) and non-influenza viruses, and predictors of influenza were identified using two different models focusing on absolute eosinopenia (0/mm^3^) and lymphocyte count <800/mm^3^. Results: Among 590 VRTIs, 116 (19.7%) were identified as outpatients, including 88 cases of influenza. Multivariable logistic regression analysis revealed the following predictors of influenza: in the first model, winter season (adjusted odds ratio [aOR] 7.1, 95% confidence interval [CI] 1.12–45.08) and absolute eosinopenia (aOR 6.16, 95% CI 1.14–33.24); in the second model, winter season (aOR 9.08, 95% CI 1.49–55.40) and lymphocyte count <800/mm^3^ (aOR 7.37, 95% CI 1.86–29.20). Absolute eosinopenia exhibited the highest specificity and positive predictive value (92% and 92.3%, respectively). Conclusion: During the winter season, specific biological abnormalities can aid physicians in identifying influenza cases and guide the appropriate use of antiviral therapy when rapid molecular tests are not readily available.

## 1. Introduction

Viral respiratory tract infections (VRTIs) are prevalent in both children and adults and often result in unnecessary antibiotic prescriptions in outpatient settings [1,2]. Moreover, excessive antibiotic prescribing can lead to inadequate treatment [3]. The 2009-H1N1 pandemic serves as an example, highlighting the importance of accurate viral species diagnosis through PCR testing to determine appropriate treatment options [4]. Currently, multiplex PCR tests are considered the most valuable diagnostic tool for VRTIs [5,6]. However, these tests can be costly, and their availability is limited in routine care for outpatients, especially outside of tertiary care hospitals. The implementation of PCR-based Point-of-Care testing in non-tertiary care settings could significantly benefit patients by providing timely and accurate diagnoses.

In contrast, blood tests that utilize costly biological markers have been extensively proposed to differentiate viral infections from bacterial superinfections. However, the two main biological markers commonly employed in clinical practice for VRTIs, namely C-reactive protein (CRP) and procalcitonin (PCT), have demonstrated their limitations over time [7,8]. Furthermore, it is worth noting that serum concentrations of CRP typically start to rise above 5 mg/L about 6 h after an infection and peak at approximately 48 h [9]. On the other hand, PCT is detectable within 3 to 4 h following an infection and reaches its peak levels at 6 to 12 h [10].

One interesting biological marker that has been repurposed during the last decades is the eosinophil count. Indeed, eosinopenia has been particularly studied during sepsis in the intensive care unit [11,12], but also as a reliable marker of in-hospital mortality among elderly during bacterial infection [13]. However, eosinopenia has not been well-established during viral infections [14], except in the case of COVID-19 as a marker of prognosis [15]. Moreover, while lymphopenia has been related to the severity of influenza [16], its clinical significance for the diagnosis of VRTIs has not been thoroughly evaluated.

It is noteworthy to consider the role of eosinophils in respiratory infections caused by viruses, such as respiratory syncytial virus (RSV). In paediatric cases of RSV infection, eosinophils are recruited to the lower airways [17]. These eosinophils contribute to virus clearance through various mechanisms, including the production of cytokines such as IFN-β that enhance host defense [18]. Similarly, studies by Samarasinghe et al. have demonstrated that eosinophils exhibit piecemeal degranulation and upregulate antigen presentation markers, promoting CD8+ T cell responses following influenza A virus challenge [19]. It is worth noting that eosinophil production is not specific to a particular type of virus and has also been observed in rhinoviruses, where eosinophils induce a T cell-virus-specific response, albeit at a different level [20]. These findings highlight the complex and diverse interactions between eosinophils and different viral infections.

Having additional tools that can assist physicians in distinguishing between different types of viruses, without incurring extra costs, presents a significant challenge, particularly in low-income countries. In light of our previous investigations on viral respiratory tract infections (VRTIs) encompassing influenza and paramyxoviruses from 2016 to 2018, which indicated no correlation between superinfection and viral species [21], we conducted an ancillary study to identify predictors of influenza infections among outpatients. The objective of this study is to offer valuable insights for initiating appropriate antiviral therapy and implementing effective isolation measures during the fall-winter season when rapid PCR testing may not be readily available.

## 2. Materials and Methods

### 2.1. Outpatients’ Selection and Objectives

This retrospective study was conducted in the Paris-Saclay hospital group, which is composed of teaching hospitals, including two main acute care facilities (Hôpital Raymond-Poincaré and Hôpital Ambroise-Paré, with 255 and 399 beds, respectively) with specialized consultation of infectious diseases and an emergency department.

Outpatients included in this study were selected from our existing cohort of patients with confirmed VRTIs using PCR, as part of the SUPERFLUOUS study (Superinfection due to inFLUenza and Other respiratory virUS), which has been described in detail elsewhere [21]. Virus identification was carried out using triplex PCRs targeting influenza A, influenza B, and respiratory syncytial virus (RSV). Additionally, separate assays were used to detect for parainfluenza viruses 1 and 3 (PIV-1, PIV-3), as well as human metapneumovirus (hMPV). These PCR assays were developed in-house and the methodology has been previously described [22].

To clarify the findings, patients’ characteristics were categorized into two groups based on the virus species detected: influenza viruses (influenza A and B) and other respiratory viruses (RSV; PIV-1, PIV-3, and hMPV). Biological abnormalities were assessed either at the emergency department or immediately after admission.

The primary objective of this study was to assess the diagnostic value of eosinophil count for influenza. A cut-off value of 0 cells/mm^3^ was used to classify the patient cohort. Eosinophil count and white blood cell count were measured using the Coulter hematology analyzer (Beckman Coulter, Fullerton, CA, USA). The secondary objective was to evaluate the significance of other predictors, including lymphocyte count, which is a commonly affected white blood cell subset in viral infections [23]. A threshold of 800/mm^3^ lymphocyte count (below or above) was used to categorize patients in the present study.

### 2.2. Statistical Analysis

Descriptive statistics are presented as counts and percentages, or means and standard deviations, with skewed continuous data summarized as medians and interquartile ranges.

Factors associated with viruses were identified using multivariable logistic regression. Potential factors included were gender, age, and factors which had a *p*-value < 0.10 in the univariate analysis and were factors identified in our previous work [21]. The fit of the model was tested using the Hosmer and Lemeshow adequacy test (*p* > 0.05).

All statistical calculations were performed using Jamovi software version 2.2.5 and R software, version 4.1.2 to assess model fit.

### 2.3. Ethics

The present work is part of the SUPERFLUOUS study performed in accordance with ethical and regulatory standards for clinical research. The study design was approved by the ethical and scientific committee for health research, studies and evaluation (CESREES), and by the Comission Nationale Informatique et Libertés (CNIL) under the number 921061.

## 3. Results

### 3.1. Study Population and Infection Characteristics

From October 2016 to June 2018, a total of 3092 tests were performed. After focusing on positive cases among outpatients, a total of 116 patients were included in the analysis. Among these patients, 88 (75.8%) were diagnosed with influenza, while 28 (24.1%) were found to have a non-influenza viral infection.

Patient characteristics are detailed in Table 1. The mean (±SD) patient age was 59 ± 10 years, with a male-female sex ratio of 0.81. The mean Charlson comorbidity index (CCI) was 3.6, considering patients with a CCI ≥ 5 are at high risk of one-year mortality.

Despite the absence of bacterial documentation, 39.6% of outpatients (*n* = 46) received antimicrobial therapy, with a mean (±SD) duration of 6.8 ± 2 days.

### 3.2. Predictors of Influenza

In the univariate analysis, age, CCI, and Fine score were associated with virus species. However, these associations were not confirmed in multivariable analysis (Table 2).

On the other hand, in the first model, absolute eosinopenia (eosinophil count = 0/mm^3^) showed a significant association with influenza (*p* = 0.03), with a specificity of 92% and positive predictive value (PPV) of 92.3% (Supplementary Table S1). In the second model, a lymphocyte count <800/mm^3^ was also found to be associated with influenza (*p* = 0.004), although with lower specificity (84%) and PPV (90.9%).

**Figure 1:**
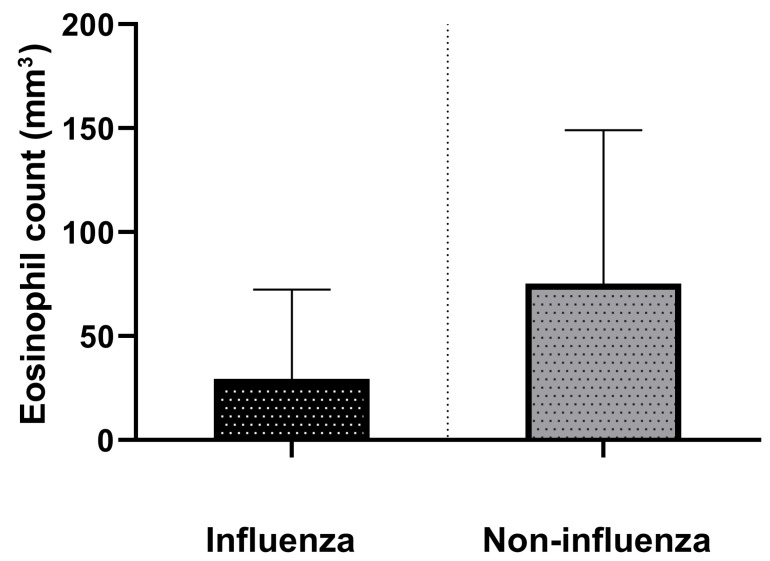
Eosinophil count distribution (mean ± SD) according to virus species.

The distribution of eosinophil count according to virus species (*p* = 0.01) is illustrated in Figure 1.

Similarly, in multivariable analysis, influenza was found to be significantly more common during the winter season compared to other viruses, depending on the model used (*p* = 0.02 and *p* = 0.04, respectively). However, the specificity was low (35.7%).

It is worth noting that treatment strategies, including the use of antibiotics, and management were similar between virus species.

## 4. Discussion

Prior to the COVID-19 pandemic, our study provided insights into the relationship between influenza and biological abnormalities among outpatients, with higher specificity and PPV for absolute eosinopenia than lymphocyte count. To the best of our knowledge, there is limited research on the evaluation of eosinophil count, specifically in the context of outpatients or primary care.

The reason why we observed a higher prevalence of absolute eosinopenia in influenza can be explained by the association of eosinopenia with the heightened systemic inflammatory response syndrome observed in influenza, whereas non-influenza viruses elicit fewer inflammatory cytokines, except for COVID-19 [24]. Furthermore, we took great care to avoid drawing the incorrect conclusion that eosinopenia is specifically associated with influenza, considering that influenza is prone to superinfection. However, the incidence of superinfection was comparable between both groups (*p* = 0.21), which mitigates the risk of overinterpretation. It is worth noting that the role of eosinophils in inflammation remains controversial; nonetheless, numerous studies conducted in animal models and humans have collectively demonstrated that eosinophils play diverse functional roles, exerting proinflammatory, inhibitory, and/or regulatory effects at sites of inflammation [25].

Although absolute eosinopenia cannot replace PCR testing for the diagnosis of VRTIs, it should be noted that PCR tests are not readily available in primary care settings for outpatients, whereas laboratory tests can be performed quickly and at no additional cost. Rapid antigen tests (RATs) may be an option in emergency departments; however, their use is limited in general practice due to lower performance compared to PCR in detecting influenza [26]. Additionally, RAT availability can be restricted in low-income countries, considering the average cost of such tests is approximately USD 20 [27]. Moreover, the utilization of RATs in Europe is largely influenced by the practices and preferences of general practitioners. Therefore, alternative tools or strategies, such as eosinopenia, that can assist physicians in distinguishing influenza from other viral species are highly valuable. This distinction can be essential for initiating specific antiviral therapy, as patients with influenza-like illness treated with oseltamivir recover approximately one day earlier on average compared to those managed with usual care [28]. It is also noteworthy that the influenza season can be reliably anticipated, even in the post-pandemic era, with a typical occurrence from October to late March in the Northern hemisphere [29]. Thus, utilizing absolute eosinophil count as an indicator for influenza, as opposed to other commonly encountered viruses, holds significant clinical relevance.

Our study also highlights the substantial burden on healthcare providers during the winter season, with a majority of consultations (86.2%) occurring during this period. This finding underscores the importance of implementing additional measures to support healthcare systems in effectively managing the seasonal surge of patients presenting with influenza-like illness symptoms, including COVID-19, RSV, and other common viruses from previous years [30].

Furthermore, 39.6% of cases received antibiotics for a VRTI, which were considered possibly secondary superinfected based on complementary investigations, despite the absence of bacterial documentation. In line with the literature, Cheysson et al. reported that VRTIs account for 40% of outpatient antibiotic use during the cold season [2]. This finding emphasizes the need for a more comprehensive antibiotic stewardship program targeting outpatients, including both emergency department settings and general practitioners. It also highlights the importance of implementing a widespread vaccination campaign to mitigate the burden of secondary bacterial infections.

Our work has several limitations. Firstly, the study was not originally designed specifically for outpatients, although a substantial number of patients initially sought care in the emergency department (*n* = 467/590) [21]. It is important to consider that the high proportion of influenza infections observed among the positive cases in our study may not be fully representative of the typical outpatient population managed by general practitioners, potentially leading to an artificially increased PPV. Indeed, a study conducted in an urban setting that focused on influenza-like illness reported a lower positivity rate of 31.2% for influenza, but it is worth noting that this study was conducted in a tropical region, introducing potential geographical variations. Secondly, due to the retrospective nature of our study, it was not possible to establish a direct correlation between biological abnormalities, such as eosinophil and lymphocyte counts, and the onset of symptoms. Thirdly, while absolute eosinopenia points towards the diagnosis of influenza, this marker exhibits low sensitivity, and some cases of influenza may have normal eosinophil counts, highlighting the need for a comprehensive diagnostic approach. Likewise, Munoz et al. reported in a cohort of COVID-19 outpatients (*n* = 249) that absolute eosinopenia accounted for the first quartile of their sample, with a median of 100/mm^3^, where he showed that an eosinophil count > 200/mm^3^ was associated with low risk of readmission [31]. Therefore, while absolute eosinopenia is a useful indicator, it should be interpreted in conjunction with other clinical and laboratory findings. Lastly, it is important to acknowledge that the resurgence of influenza during the winter of 2023 in Europe, surpassing even the prevalence of COVID-19, highlights the significance of absolute eosinopenia as a diagnostic marker. However, the generalizability of our findings to other regions or future influenza seasons should be considered cautiously [29].

Despite these limitations, our study provides valuable insights into the potential utility of absolute eosinophil count in the diagnosis of influenza among outpatients, especially in the context of the ongoing resurgence of influenza and the challenges posed by COVID-19. Further prospective studies are warranted to validate and refine the use of this marker in clinical practice.

In conclusion, our study highlights the importance of implementing new diagnostic tools for outpatients, aiming to reduce unnecessary antibiotic prescriptions in cases of typical VRTIs [21]. This represents a notable advancement compared to the time our study was conducted, as these tests have now become widely accessible beyond tertiary care hospitals, particularly in light of the ongoing pandemic.

Our findings reveal a robust association between virus species, specifically influenza, and eosinophil and lymphocyte counts among outpatients. The simplicity of interpreting absolute eosinopenia as a helpful indicator for physicians in the absence of molecular tests holds particular value, especially in resource-limited settings. Further research on eosinopenia in the context of other viral respiratory tract infections is warranted, considering the global spread of COVID-19 and the increasing availability of new molecular testing methods to confirm these preliminary findings.

## Figures and Tables

**Table 1:** Outpatient characteristics (*n* = 116).

Baseline Characteristics	Value
Age, mean (SD), y	59 (10)
Male sex, *n* (%)	52 (44.8)
Chronic respiratory disease, *n* (%)	23 (19.8)
CCI * < 5, *n* (%)	73 (62.9)
Fine score, mean (SD)	65.7 (33.3)
**Management**	
Consulting an ER physician, *n* (%)	88 (75.8)
**Period**	
Season 2016–2017, *n* (%)	40 (34.5)
Season 2017–2018, *n* (%)	76 (65.5)
Fall, *n* (%)	7 (6.0)
Winter, *n* (%)	100 (86.2)
**Biology and imaging (*n* = 96)**	
PMN count ≥ 7000/mm^3^, *n* (%)	20 (20.8)
Lymphocyte count < 800/mm^3^, *n* (%)	44 (45.8)
Eosinophil count = 0/mm^3^, *n* (%)	26 (27.1)
Radiological abnormalities	10 (10.4)
**Treatment strategies**	
Antimicrobial therapy initiated for superinfection, *n* (%)	46 (39.6)
Treatment duration, mean (SD)	6.8 (2)

* CCI, The Charlson Comorbidity Index ≥ 5 reflects patient’s fragility.

**Table 2:** Potential factors associated with influenza infection: logistic model regression.

Variables	Univariate Model		Multivariable Model 1		Multivariable Model 2	
	OR [IC95%]	*p* Value	aOR [IC95%]	*p* Value	aOR [IC95%]	*p* Value
			Adjusted on Age, Sex, Eosinophil Count		Adjusted on Age, Sex, Lymphocyte Count	
**Baseline characteristics**						
Age (years)	0.97 [0.95–0.99]	0.01	0.96 [0.91–1.01]	0.12	0.95 [0.89–1.01]	0.11
Sex (male)	0.90 [0.38–2.12]	0.81	-	-	-	-
Chronic respiratory disease	0.66 [0.24–1.83]	0.43	-	-	-	-
CCI * < 5	3.01 [1.25–7.23]	0.01	0.30 [0.07–1.26]	0.30	0.29 [0.07–1.21]	0.09
Fine score (mean)	0.99 [0.97–1.00]	0.04	1.02 [0.97–1.05]	0.46	1.02 [0.98–1.05]	0.30
**Management**						
Consulting an ER physician	1.36 [0.52–3.55]	0.53	-	-	-	-
**Period**						
2016–2017 vs. 2017–2018	1.44 [0.76–2.74]	0.26	-	-	-	-
Season: Spring	Reference		Reference		Reference	
Fall	0.5 [0.06–4.09]	0.51	0.52 [0.04–7.28]	0.62	0.95 [0.07–13.47]	0.97
Winter	5.69 [1.38–23.33]	0.02	7.1 [1.12–45.08]	**0.04**	9.08 [1.49–55.40]	**0.02**
**Biology and imaging**						
PMN count ≥ 7000/mm^3^	0.43 [0.15–1.23]	0.12	-	-	-	-
Lymphocyte count <800/mm^3^	6.77 [2.11–21.77]	0.001	-	-	7.37 [1.86–29.20]	**0.004**
Eosinophil count = 0/mm^3^	5.87 [1.28–27.05]	0.02	6.16 [1.14–33.24]	**0.03**	-	-
Radiological abnormalities	0.72 [0.17–2.99]	0.65	-	-	-	-
**Treatment strategies**						
Antitbiotics for superinfection	0.58 [0.25–1.38]	0.21	-	-	-	-
Treatment duration (mean)	0.94 [0.84–1.06]	0.36	-	-	-	-

OR, Odds ratio; aOR, adjusted Odds ratio; CI, confidence interval; NS, not significant (*p* > 0.05); * CCI, The Charlson Comorbidity Index ≥ 5 reflects patient’s fragility; PMN, polymorphonuclear neutrophils; ER, emergency room. Multivariable logistic regression was used to identify the potential factors associated with the type of virus, adjusted for age, sex, and for variables with a *p* < 0.10 in univariate analysis. The adequacy test used is an adjustment by Hosmer and Lemeshow.

## Data Availability

On request.

## Supplementary Materials

The following supporting information can be downloaded at: https://www.mdpi.com/article/10.3390/diagnostics13122115/s1, Supplementary Table S1: Performance of eosinophil count and lymphocyte count to diagnose influenza infection among ambulatory care individuals suffering from viral respiratory tract infections.
