# Supplementary material for: Interest of Absolute Eosinopenia as a Marker of Influenza in Outpatients during the Fall-Winter Seasons 2016–2018 in the Greater Paris Area: The SUPERFLUOUS Study
Source: Diagnostics (Basel). 2023 Jun 19;13(12):2115. doi: 10.3390/diagnostics13122115 (PMC10296893; doi:10.3390/diagnostics13122115)
Supplement: Supplementary file 1 [file diagnostics-13-02115-s001.zip › diagnostics-2346063-supplementary.pdf]

Supplementary Table S1. Performance of eosinophil count and lymphocyte count to diagnose influenza infection among ambulatory care individuals suffering from viral respiratory tract infections

Parameter	Sensitivity	Specificity	Predictive Positive Value	Negative Predictive Value
**Eosinophil count = 0 /mm^3**	33.8%	92%	92.3%	32.9%
**Lymphocyte count <800/mm^3**	56.3%	84%	90.9%	40.4%
**Winter season**	93.2%	35.7%	82%	62.5%
**Eosinopenia=0/mm^3 during winter**	34.8%	87.5%	92%	24.5%
